# Application of Walnut Shells-Derived Biopolyol in the Synthesis of Rigid Polyurethane Foams

**DOI:** 10.3390/ma13122687

**Published:** 2020-06-12

**Authors:** Sylwia Członka, Anna Strąkowska, Agnė Kairytė

**Affiliations:** 1 Institute of Polymer and Dye Technology, Faculty of Chemistry, Lodz University of Technology, Stefanowskiego 12/16, 90-924 Lodz, Poland; anna.strakowska@p.lodz.pl; 2 Faculty of Civil Engineering, Institute of Building Materials, Laboratory of Thermal Insulating Materials and Acoustics, Vilnius Gediminas Technical University, Linkmenu st. 28, LT-08217 Vilnius, Lithuania; agne.kairyte@vgtu.lt

**Keywords:** Polyurethanes, walnut shells, bio-polyol, insulating properties, mechanical properties

## Abstract

This study aimed to examine rigid polyurethane (PUR) foam properties that were synthesized from walnut shells (WS)-based polyol. The Fourier Transform Infrared Spectroscopy (FTIR) results revealed that the liquefaction of walnut shells was successfully performed. The three types of polyurethane (PUR) foams were synthesized by replacement of 10, 20, and 30 wt% of a petrochemical polyol with WS-based polyol. The impact of WS-based polyol on the cellular morphology, mechanical, thermal, and insulating characteristics of PUR foams was examined. The produced PUR foams had apparent densities from 37 to 39 kg m^−3^, depending on the weight ratio of WS-based polyol. PUR foams that were obtained from WS-based polyol exhibited improved mechanical characteristics when compared with PUR foams that were derived from the petrochemical polyol. PUR foams produced from WS-based polyol showed compressive strength from 255 to 310 kPa, flexural strength from 420 to 458 kPa, and impact strength from 340 to 368 kPa. The foams that were produced from WS-based polyol exhibited less uniform cell structure than foams derived from the petrochemical polyol. The thermal conductivity of the PUR foams ranged between 0.026 and 0.032 W m^−1^K^−1^, depending on the concentration of WS-based polyol. The addition of WS-based polyol had no significant influence on the thermal degradation characteristics of PUR foams. The maximum temperature of thermal decomposition was observed for PUR foams with the highest loading of WS-based polyol.

## 1. Introduction

Polyurethanes (PUR) are one of the most important classes of polymeric materials and they are widely used in different applications, such as furnishing, packaging, and building construction [1,2]. PUR are synthesized from two basic compounds—polyols and isocyanates [3,4]. The main concern that is connected with PUR materials is their petroleum-dependent synthesis, since both polyols and isocyanates are petroleum-derived [3]. The development of bio-based compounds has attracted great interest due to environmental issues and fluctuations in oil prices. Many studies have demonstrated that several directions can be taken to make an eco-friendlier production process of PUR, and all of them are strongly encouraged. For example, the preparation of bio-polyols that are based on different kinds of oils, such as canola oil [5], sunflower oil [6], and rapeseed oil [7,8], have already been reported.

Other renewable resources applied for the production of polyols constitute lignocellulosic biomass. When comparing to bio-polyols derived from plant oil, the main advantage of lignocellulosic is their non-food use; therefore, the application of lignocellulosic materials for the production of bio-polyols does not compete with food aspect [9,10]. The chemical composition of lignocellulosic compounds mainly involves cellulose, hemicellulose, and lignin, which contain two or more –OH groups per molecule. Due to this, the liquefaction of lignocellulosic compounds is considered a great resource for the production of PUR materials [11,12]. Bio-polyols derived from some kinds of lignocellulosic compounds, such as sawdust [13], cotton [14], bamboo [15], bagasse [16], or jute fibers [10], have already been reported in previous studies. The results have shown that the application of bio-polyols in the production of PUR foams results in materials with comparable mechanical, thermal, and insulating properties to those that were synthesized from the petrochemical polyol. 

For example, lignin-based PUR foams were produced by Xue et al. [17]. PUR foams based on the bio-polyol derived from liquefied corncob were additionally reinforced with pulp fibers. The results have shown that, when compared to PUR foams based on petroleum-derived polyols, the obtained composites exhibit lower apparent density and larger cells, due to the presence of pulp fibers that are embedded in the foam structure. Furthermore, the addition of bio-polyol resulted in the improvement of thermal and insulating characteristics of PUR foams. Interesting results were also reported by Ertas et al. [18] in the case of PUR foams that are based on liquefied eucalyptus and pine woods compounds. The liquefaction process was performed in a mixture of glycerin and polyethylene glycol. The examined properties were found to be comparable with those that were obtained for PUR foams derived from the petrochemical polyol. The mechanical and thermal properties of bio-based PUR foams were in line with other synthetic-based foams. Similar results were obtained by Domingos et al. [19]. The authors synthesized PUR foams based on liquefied eucalyptus branches. It has been shown that the application of bio-polyol for the production of PUR foams might be a successful approach for obtaining PUR materials with comparable thermal properties, although with slightly worsened mechanical characteristics. Kosmela et al. prepared marine-biomass derived polyols [20]. The replacement of petroleum-derived polyol with up to 30 wt% of bio-polyol increased the reactivity of the polyol system. Due to this, the obtained PUR foams exhibited improved mechanical, thermal, and insulating characteristics when compared with synthetic PUR foams. 

Bio-polyol that was obtained from cashew nut shells was successfully used in making rigid PUR foams with good mechanical, thermal, and fire properties by Ionescu et al. [21]. Similar results were reported by Gandhi et al. [22]. Bio-polyol obtained from cashew nut shell liquid derived through cashew processing was selected as an optimal candidate to synthesize PUR foam with enhanced and functional properties. Interesting results were reported by De Luca Bossa et al. [23]. Sustainable nanocomposite PUR foams were successfully produced starting from Cardanol-based polyol and additionally enhanced with three natural fillers—walnut shell, cellulose, and diatomite. The results showed that, with respect to the pristine PUR foam, walnut shell, and cellulose, through their OH groups, react with isocyanate precursor producing PUR foams with an improvement in the thermal stability and mechanical properties. 

The presented results confirmed that lignin derived from chemical pulping processes and biorefining processes are promising candidates for use in PUR synthesis if well-established challenges can be overcome. In addition to increasing the bio-based content of the polyurethane products, lignin incorporation into various PUR products has been shown in some cases to provide performance advantages that include enhanced crosslinking density, improved biodegradability, increased ultraviolet (UV) stability, antioxidant properties, and improved mechanical strength and thermal stability of the final product [24]. Besides many beneficial aspects of lignin-based polyols, several challenges have been identified for the replacement of conventional polyols with lignins that have limited its utility in PUR. Chung et al. [25] reported that the reactivity of hydroxyl groups within lignin towards an isocyanate might be restricted due to the steric hindrance as a consequence of both the higher order structure and, potentially, self-association of the lignin polymer that limits access to hydroxyl groups. Lignin solubility is another problem for many PUR products. Lignins must be soluble in the solvent used for the reaction or with other polyols used in the synthesis, which might be a challenge for some applications. Lignins that are derived from the alkaline delignification of grasses, hardwoods, and softwoods are partially or completely soluble in many organic solvents, with many lignins exhibiting the general trend of increasing solubility with increasing solvent polarity in organic solvents [26]. Besides, many types of lignin may contain sulfur that contributes to odor problems in final products, while product yellowing due to sulfur can be problematic in some polymer applications [27]. Removing sulfur, particularly covalently bound sulfur, might not be an economically feasible solution. Another problem for lignin-based PUR is UV instability connected with unreacted phenolic hydroxyl groups of lignin. It has been stated that, at high loadings, lignin was demonstrated to contribute to the photodegradation process [28,29,30]. Most of these challenges have been identified for unfractionated, unfunctionalized kraft lignins. Lignins from other sources and other processing approaches, subjected to different purification and potentially subjected to chemical modifications, such as functionalization or depolymerization, represent potential pathways for integrating novel lignins into PUR products.

The worldwide development and growth in the applications of bio-based PUR foams lead to the necessity for finding and establishing new sources of bio-polyols. Among different organic materials, solid residues of plants that consist of lignin, cellulose, and hemicellulose may be considered as sustainable liquefied bio-polyol for the synthesis of PUR foams. An example of such material is walnut shells (*Juglans regia* L.). Walnut is an important plant that is cultivated to obtain its edible part of the fruit, i.e., nuts [31]. Because walnuts are mainly grown to obtain kernels, other parts of the fruit, such as husks and shells that are producing during fruit harvesting and processing are considered as waste materials [32]. Taking into consideration that the composition of walnut consists of 67% of shells, during the walnut harvesting around 1.5 tons of walnut shells are produced and left behind every year [33]. Walnut shells can be utilized for the production of high-value bio-polyols for the production of a new class of PUR foams due to their rich organic nature (lignin ~50%, cellulose ~24%, hemicellulose ~24%), and low ash content (3.4%) [34,35]. When compared to other organic materials, the main advantages of lignocellulosic walnut shells are their large availability around and renewable nature. When considering that bio-polyols obtained from liquefied walnut shells have not been applied in the production of PUR foams to date, it seems logical and fully justified to use walnut shells in the production of PUR composites. When taking into consideration that the importance of walnut shells on environmental pollution the production of PUR foams from liquefied walnut shells might have a beneficial impact on the economy of PUR foams and successfully solve the problem connected with the utilization of walnut shells. 

## 2. Experimental

### 2.1. Materials

Two-components system (STEPANPOL PS-2352—polyester polyol with a hydroxyl number of 240 mgKOH/g and PUROCYN B—polymeric diphenylmethane-4,4′-diisocyanate) was provided by Purinova Sp. z o.o.(Bydgoszcz, Poland);

- Kosmos 33 (potassium acetate) and Kosmos 75 (potassium octanoate) purchased from Evonik Industries AG (Essen, Germany) were selected as catalysts;
- TEGOSTAB B8513 purchased from Evonik Industries AG was used as a silicone surfactant;
- Cyclopentane (purity > 98%) and Pentane (purity ≥ 99%) provided by Merck KGaA (Darmstadt, Germany) were used as a blowing agent;
- Polyethylene glycol PEG-400 (purity 95%), sodium hydroxide (anhydrous), phthalic anhydride (purity ≥ 99%), glycerol (purity > 99.5%), imidazole (purity ≥ 99%), sulfuric acid (purity 95–98%), and pyridine (purity ≥ 99.8%) were purchased from Sigma–Aldrich Corporation(St. Louis, MO, USA); and,
- Walnut shells (WS) were obtained from a local company from Poland.

### 2.2. Methods

#### 2.2.1. Liquefaction of WS

The walnut shells were dried at 100 °C for 48 h, ground in a knife mill, and finally sieved using a 50 mesh fraction. Afterward, the liquefaction of WS was performed in a 250-mL flask equipped with an overhead mechanical stirrer. 12 g of WS, 96 g of PEG 400, and 24 g of glycerol were poured into the flask and then heated in an oil bath with a temperature of 160 °C. Subsequently, 2 g of sulfuric acid was poured into the mixture within 30 min. with constant stirring. The temperature was kept constant at 160 °C and the mixture was stirred for another 3 h. Such obtained sulfuric mixture was neutralized with sodium hydroxide to pH of 6–7. The liquefied mixture was cooled down and separated using a Buchner funnel, while the liquefaction residue was washed by pure, deionized water. The liquefaction residue was dried at 100 °C for 48 h and the liquefaction yield was calculated following Equation (1).

(1) $$\mathrm{Liquefaction}\;\mathrm{yield}\;\left[\%\right]=\left(1-\frac{\mathrm{Solid}\;\mathrm{residue}\;\left[g\right]}{\mathrm{Dry}\;\mathrm{WS}\;\left[g\right]}\right)\cdot 100\%$$

#### 2.2.2. PUR Foams Preparation

**Figure 1:**
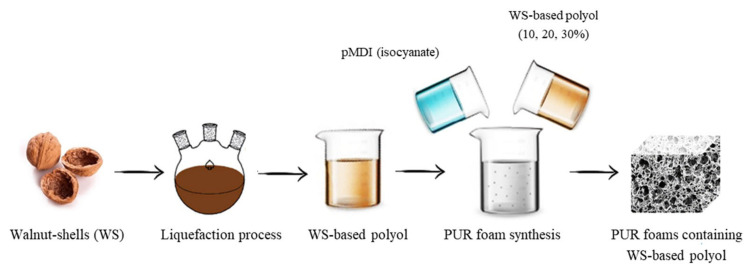
Schematic procedure for preparing PUR foams.

PUR foams were prepared in open forms. The calculated amounts of petrochemical polyol, WS-based polyol, catalysts, surfactant, physical blowing agent, and flame retardant were placed in an open form and intensively mixed with a mechanical stirring with the constant rate of 1000 rpm (round per minute) over 30 s in order to obtain a homogenous blend. Subsequently, pMDI was added and the obtained mixture was stirred for another 30 s. The pMDI was used in the ratio of 2:1 in relation to the hydroxyl groups. The form was placed on a flat surface and the resulting mixture was expanded vertically. The obtained foams were conditioned in the room temperature for 24 h. Table 1 presents all of the formulations of PUR foams. Figure 1 presents the schematic procedure of PUR foam preparation.

### 2.3. Methods

The viscosity of WS-based polyol and petrochemical polyol was determined following ISO 2555. The measurement was performed using Viscometer DVII+ (Brookfield, Berlin, Germany) at a constant shear rate of 10 rpm. The viscosity was evaluated at room temperature. 

The hydroxyl number of the WS-based polyol was determined based on ISO 14900. Phtalation reagent was prepared by mixing phthalic anhydride (110 g) and imidazole (17 g) in pyridine (700 mL). WS-based polyol (1 g) was added to the phtalation reagent and such a prepared mixture was heated up to 100 °C for 30 min. After this time, the pyridine (50 mL) and distilled water (10 mL) were added to the mixture. Finally, the mixture was titrated with KOH solution (0.5 M) and the hydroxyl number was calculated according to Equation (2):

(2) $$\mathrm{Hydroxyl}\;\mathrm{number}=\frac{\left(A-B\right)\left(56.1\right)\left(N\right)}{W}$$

where, A and B refer to the consuming volume (mL) of the KOH at the neutralization point for a blank test (no polyol) and analyzed sample (WS-based polyol), respectively; N refers to the normality of KOH solution and W is a sample weight (g).

The chemical structure of WS-based polyol, petrochemical polyol, and PUR foams were evaluated by means of Fourier Transform Infrared Spectroscopy (FTIR). The analysis was performed using Nicolet iS50 spectrometer (Thermo Fisher Scientific, Waltham, MA, USA). 

Liquid-state ^1^H NMR spectra were obtained with a Bruker (Billerica, MA, USA) Avance 500 NMR spectrometer (USA). The acetylated lignin and lignin polyol samples (150 mg) were dissolved in dimethyl sulfoxide (DMSO-d6) (0.40 mL). Spectral analyses were performed while using Bruker software (TopSpin software, 4.0, Billerica, MA, USA).

The apparent density of PUR foams was determined following ISO 845. The apparent density was determined as the weight to volume ratio of the PUR foams.

The cellular morphology of PUR foams was examined by means of scanning electron microscope (SEM) using JSM-5500 LV (JEOL Ltd., Peabody, MA, USA) at the accelerating voltage of 10 kV. Each sample was tested in the free-rise direction.

The closed-cells content was determined following the ISO 4590 standard.

The thermal conductivity of PUR foams was examined using the heat flow meter with a brand name of LaserComp 50 (HFMA, Westchester, IL, USA). 

Compressive strength (*σ*_10%_) was determined following the ISO 844 standard. The measurement was performed using Zwick Z100 Testing Machine (Zwick/Roell Group, Ulm, Germany). The PUR samples were compressed up to 10% of deformation in the parallel and perpendicular direction to the PUR foam rise direction under the load speed of 2 kN. 

The flexural strength (*σ_f_*) and impact strength (*σ_I_*) of PUR foams were determined following ISO 178 and ISO 180 standards. The measurements were performed using Zwick Z100 Testing Machine (Zwick/Roell Group, Germany) at a constant speed of 2 mm min.^−1^. 

Dynamic mechanical analysis (DMA) was performed for samples with 2 mm of thickness. The measurement was performed using ARES Rheometer (TA Instruments, New Castle, DE, USA). The PUR samples were tested in the temperature range from 0 to 250 °C. The applied deformation was 0.1% and the used frequency was 1 Hz.

Thermogravimetric analysis (TGA) was applied to determine the thermal characteristics of PUR foams. The measurement was performed using STA 449 F1 Jupiter Analyzer (Netzsch Group, Selb, Germany). The samples were tested in the temperature range from 0 to 600 °C

The color change of PUR samples was determined using a CM-3600 d spectrophotometer (Konica Minolta Sensing, Tokyo, Japan). The samples were tested in the wavelength range between 360 and 740 nm.

The water uptake of the PUR samples was determined following ISO 2896. The water uptake was calculated according to Equation (3), where m_0_ refers to the initial mass of the sample (before immersing in distilled water) and m refers to the mass of the sample after immersing in 1 cm of distilled water (the samples were immersed for 72 h).

(3) $$WA=m-m_{0}/m_{0}\;\;$$

The dimensional stability of PUR foams after conditioning at the temperature of −20 °C and +70 °C was determined according to ISO 2796. The PUR samples were conditioned for 14 days. 

## 3. Results and Discussion

### 3.1. Characterization of WS-Based Polyol

Table 2 presents the results of viscosity, hydroxyl number, water content, and average molecular weight (M_w_) of WS-based polyol and petrochemical polyol (Stepanpol PS-2352). The viscosity, hydroxyl number, water content, and molecular weight of WS-based polyol are 2550 mPa·s, and 340 mgKOH/g, 0.8%, and 420 Da, respectively. The WS liquefaction ratio is determined as 87.2%. All of the above values are in the range of those reported previously [36].

**Figure 2:**
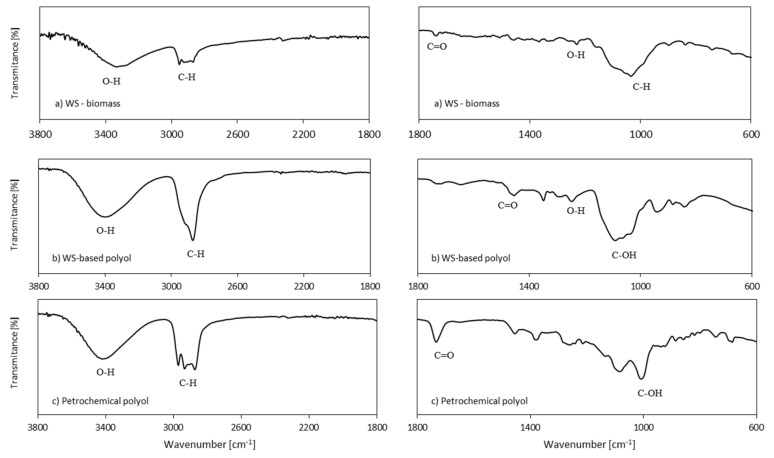
Fourier Transform Infrared Spectroscopy (FTIR) analysis of (**a**) WS-biomass, (**b**) WS-based polyol, and (**c**) petrochemical polyol.

WS and WS-based polyol were evaluated by FTIR analysis to understand changes in functional groups which occur during the liquefaction process. The FTIR spectra of WS and WS-based polyol are presented in Figure 2b,c, respectively. Intense bands at 1030 and 2955 cm^−1^ refer to the stable bands of cellulose, hemicellulose, and lignin of WS, and they are attributed to the C-H vibration [37,38,39,40]. The band located at 1735 cm^−1^ refers to the C=O vibration of holocellulose [41]. A wide band ranged from 3500 to 3300 cm^−1^ refers to the hydroxyl groups of lignin [42]. The band related to OH groups is also presented on the FTIR spectrum of the WS-based polyol, which indicates a great number of hydrogen groups. Band located at 2860 cm^−1^ refers to C-H stretching vibration (-CH, -CH_2_, -CH_3_ groups) [43]. The marked increase in this peak intensity can be attributed to the structure of PEG and glycerol and could indicate that the liquefaction reaction is caused by a successful chain extension reaction that converts WS into polyol. The band at 1455 cm^−1^ refers to C=O groups and indicates the dissociation of ether bonds during the liquefaction process [43,44]. The band that is located at 1100 cm^−1^ indicates the presence of ether and C-OH groups [45]. The shift of small band from 1240 cm^−1^ (in WS) to 1210 cm^−1^ (in WS-based polyol) confirms the reaction of OH groups of aromatic units of lignin with liquefaction solvents in WS-based polyol [43,46]. The band of the carbonyl group in esters at 1740 cm^−1^ is not observed, which indicates that the product is a polyether polyol. Based on FTIR results, it is obvious that, during the liquefaction process, the WS was successfully degraded and dissolved in the used solvents. These FTIR results for polyol products are in agreement with reports of other researchers for liquefaction of agricultural residues of biomass [47]. Figure 2c presents the FTIR spectrum of the petrochemical polyol. The appearance of the characteristic carbonyl group (C=O) between 1750 and 1650 cm^−1^ confirms the existence of the ester structure of the polyol ester [48].

**Figure 3:**
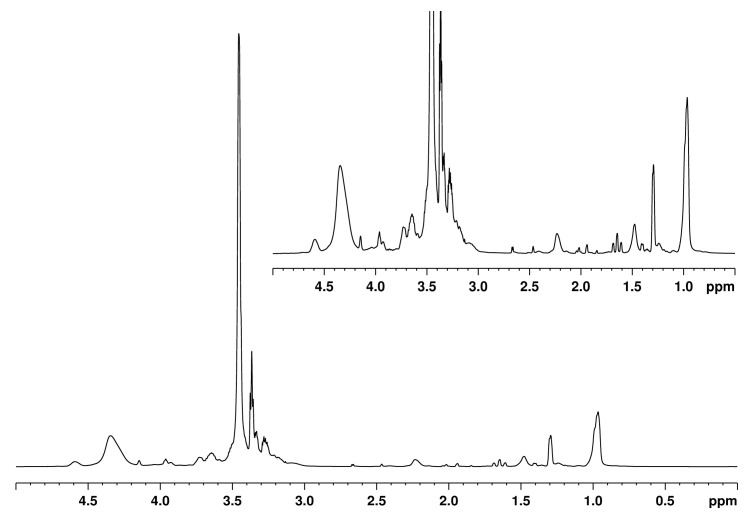
^1^H NMR spectra of WS-based polyol.

The chemical structure of WS-based polyol products was also examined by NMR spectroscopy. Figure 3 shows the ^1^H NMR spectra of WS-based polyol. The signal at 2.3 ppm is indicative of protons in dimethyl sulfoxide (DMSO), which was used as a deuterated solvent in NMR analysis. Signals between 1.5 and 0.8 ppm can be assigned to the aliphatic moiety of lignin [46]. Signals between 2.4 and 2.2 ppm refer to aromatic acetyl groups. An intense signal located at 3.4 ppm confirms the presence of polyethylene glycols, glycerol, and ether linkages (C-O-C). The peaks near between 3.8 and 3.6 ppm can be assigned to methyl groups [49]. The peaks that were observed at between 4.5 and 4.2 ppm correspond to protons in hydroxyl groups of the lignin polyols, which confirm the successful liquefaction of WS.

### 3.2. FTIR Analysis of PUR Foams

**Figure 4:**
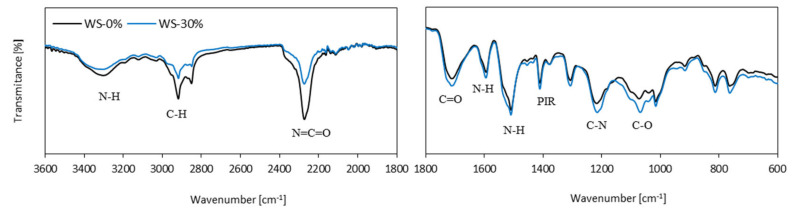
FTIR analysis of PUR foams.

The FTIR spectra of PUR foams are extremely similar, as shown in Figure 4. The urethane bonds of PUR are well represented by the absorption bands located at 1510 and 1595 cm^−1^, which refer to *δ*(N-H) [50]. Other bands that are characteristic for PUR bonds are located at 1770–1700 cm^−1^ and 3600–3200 cm^−1^, and they correspond to ν(N-H) and *δ*(N-H) [51,52]. 

When compared to neat WS-0%, the spectra of PUR foams modified with the addition of WS-based polyol are characterized by the same location of the main, characteristic bands. Due to this, it might be concluded that the addition of WS-based polyol has no influence on the chemical structure of PUR foams. By comparing the spectra, only insignificant differences can be found in the intensity of the absorption bands. As the weight ratio of the WS-based polyol increases, the intensity of the band at 1725 cm^−1^, attributed to the free urethane carbonyl group ν(C=O), also increases. This might be connected with a higher value of hydroxyl number of WS-based polyol when compared with petrochemical polyol (340 vs. 230–250 mg KOH/g). The incorporation of WS-based polyol results in the reduced intensity of the band located at 2270 cm^−1^ that corresponds to the residual isocyanate groups *v*(-N=C=O) [50]. By increasing the WS-based polyol content, the intensity of the band slightly decreases. The reduced intensity of the band located around 1100 cm^−1^ can be ascribed to the C-N urethane linkages due to the reaction between the NCO groups and OH groups of lignin leading to the formation of the new lignin-based PUR foams.

### 3.3. Foaming Kinetic of PUR Foams

The replacement of petrochemical polyol with 10–30% of WS-based polyol influences the foaming kinetics of PUR systems. The foaming process was evaluated by monitoring start, growth, and tack-free time. Table 3 presents the results.

**Figure 5:**
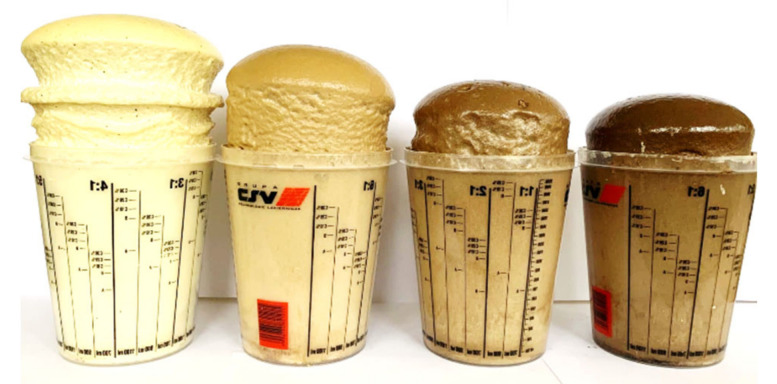
PUR foams obtained in the study: (**a**) WS-0%, (**b**) WS-10%, (**c**) WS-20%, and (**d**) WS-30%.

The start and growth time have increased by increasing the weight ratio of WS-based polyol. Previous studies have shown that the growth time is associated with the production of blowing agents (CO_2_) and the expansion of cells as a result of CO_2_ diffusion into the gas bubbles [43,53,54]. Increasing the growth time can result from two combined effects: higher hydroxyl number of WS-based polyol as compared to petrochemical polyol (340 vs. 250) results in a greater number of hydroxyl groups being able to react with isocyanate groups (-NCO). Therefore, by increasing the weight ratio of WS-based polyol in PUR systems, the number of NCO groups available to react with water is reduced, which results in a limited generation of CO_2_. Moreover, in the case of PUR foams containing WS-based polyol, the expansion of bubble cells is hindered by higher viscosity when compared to WS-0%. The mass transfer of CO_2_ from the solid to the gas phase is limited and the expansion of the PUR foam is reduced due to the increase in the viscosity of PUR systems [43,53,54]. On the other hand, the higher viscosity of WS-based polyol limits the mobility of the polymeric chains slowing down the polymerization of PUR [55]. This is also manifested by a reduced value of maximum temperature (T_max_) during the synthesis of PUR (Table 3). By increasing the weight ratio of WS-based polyol, the value of T_max_ decreases by 17, 20, and 30% for WS-10%, WS-20%, and WS-30%, respectively. Furthermore, due to the limited expansion of the cells, the height of the PUR foams is reduced (Figure 5)—e.g., by increasing the weight ratio of WS-based polyol up to 30% the height of WS-30% is reduced by ~30% when compared with WS-0%.

### 3.4. Apparent Density of Pur Foams

By increasing the weight ratio of WS-based polyol, the density of PUR foams increases, as shown in Table 4. A possible explanation may be found in the chemical structure of WS-based polyol. Due to the presence of the benzene ring of lignin, the structure of the resulting PUR is less soft and resilient compared to PUR based on petrochemical polyol. The hardness of hard and soft segments of PUR foams synthesized from WS-based polyol may be greater when compared with PUR that is formed by petrochemical polyol. The second effect is related to the reduced CO_2_ generation and limited expansion of PUR foams. Therefore, smaller cells are created and, subsequently, the density increases from ~36 kg m^−3^ (WS-10%) to ~39 kg m^−3^ (WS-30%). Similar results can be found in other research. For example, Kosmela et al. [20] observed an increase in the apparent density in the case of PUR foams synthesized from the marine biomass-derived polyol. The addition of 30 wt% of synthesized bio-polyol insignificantly increased the density from 49.2 to 50.8 kg m^−3^.

### 3.5. Cellular Morphology of PUR Foams

**Figure 6:**
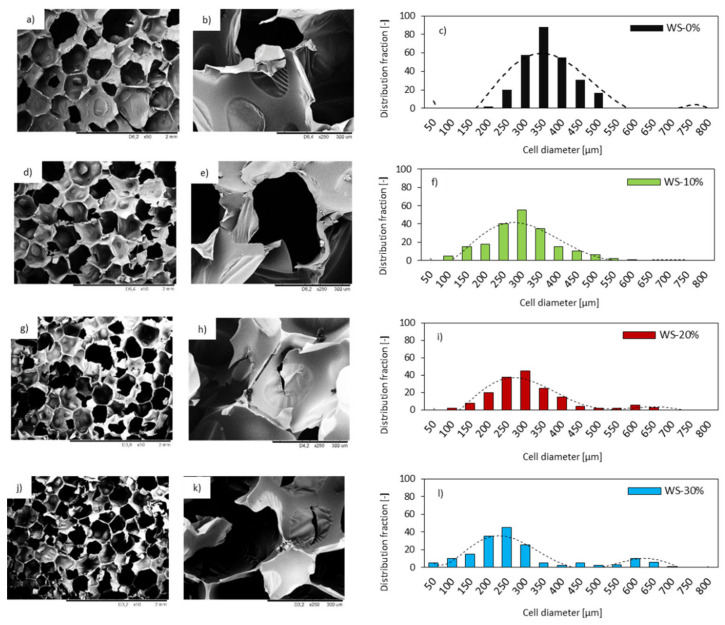
Morphology and cell size distribution of (**a**–**c**) WS-0%; (**d**–**f**) WS-10%; (**g**–**i**) WS-20%; and, (**j**–**l**) WS-30%.

The cellular structure of PUR foams has been investigated since the mechanical properties of the composites are influenced by the morphology. The cellular structure is an important parameter in controlling the mechanical and thermal properties of PUR foams. Figure 6 provides the SEM images of PUR foams with the various weight ratio of WS-based polyol.

Figure 6 shows that, with increasing the WS-based polyol content, the regularity of PUR foams slightly decreases. Additionally, there is a little change between the WS-0% and the WS-based PUR foams regarding their cell size. The cell size of WS-based PUR foams appears to be reduced when compared with the WS-0% and the distribution of the cell size is less homogeneous. The foam WS-10% exhibits cell sizes of 320 ± 8 μm diameters, whereas foams WS-20% and WS-30% provide cell sizes of 310 ± 6μm and 280 ± 8 μm, respectively. Moreover, PUR foams containing 20 and 30% of WS-based polyol (WS-30%) exhibit a wider range of cells and a lower frequency of cell size distribution when compared with the neat WS-0%. 

**Figure 7:**
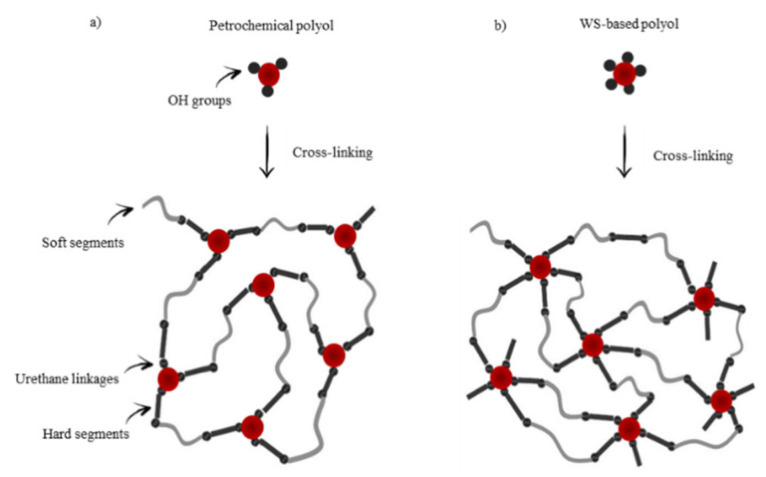
Molecular structure of PUR foams based on (**a**) petrochemical and (**b**) WS-based polyol.

Morphological changes of PUR foams may be connected with higher reactivity of WS-based polyol when compared with a petrochemical polyol, due to its higher hydroxyl number. Additional hydroxyl groups of WS-based polyol act as a cross-linking point and they can increase the cross-linking density and result in a more branched structure of PUR foams (Figure 7). However, it should be also concerned, that when the WS-based content is higher, such as 30%, an excess isocyanate can react with the hydroxyl groups in WS-based polyol and lead to more little molecular structures rather than the three-dimensional network. In turn, this might disintegrate the PUR structure and promote the formation of open cells.

The incorporation of a higher number of OH groups might also result in a higher consumption of NCO groups, as discussed previously. As a result, the reduced number of NCO groups can react with water, thereby reducing the production of CO_2_. Therefore, the expansion of the cells is hindered. Besides, an insignificant decrease in cell size can be attributed to solid particles of WS that are introduced in the WS-based polyol (WS conversion is ~87.2%). The incorporated WS’s particles can act as nucleation sites, thereby reducing the average diameter of the cells. Some of WS’s particles are visible in the cell struts, as presented in Figure 6. Therefore, with increasing the weight ratio of WS-based polyol in the PUR system, the structure with the presence of smaller cells is formed. 

By increasing the weight ratio of WS-based polyol from 10 to 30%, the number of closed-cell decreases from 89 to 80%. The possible explanation of the reduced number of closed-cells might be found in interphase interaction between WS particles and PUR matrix, which disrupts the foaming process and results in the formation of a more defective structure. It has been shown on SEM images that WS particles are not completely built in the PUR matrix, but some bigger aggregates are located in empty cells. This indicates poor interfacial adhesion between the WS surface and the PUR matrix, which, in turn, leads to cell collapse and the formation of open pores in the foam structure.

### 3.6. Thermal Conductivity of Pur Foams

The thermal conductivity (λ) is a crucial factor that determines the application of PUR foams as thermal insulation materials [56,57,58]. According to the National Standards established, materials are classified as insulating materials, if the value of the thermal conductivity coefficient is below 0.030 W m^−1^K^−1^ [59]. The value of λ of PUR foams was calculated based on the following equation: λ = λ_s_ + λ_g_ + λ_c_ + λ_r_ (where λ_s_ corresponds to the λ through the solid phase, λ_g_ refers to the λ of the gas in the foam’s cells, λ_c_ is a λ connected with the convection across the cells, and λ_r_ refers to radiation through the cells) [60]. Thermal conductivity for neat WS-0% is 0.0.24 W m^−1^K^−1^, as presented in Table 4. The value of λ increases with increasing the weight ratio of WS-based polyol. For WS-30% the value of λ increases from 0.024 to 0.032 W m^−1^K^−1^, which is 33% higher than that of the neat WS-0%. Previous studies have shown that the λ of PUR foams depends on the cellular morphology of PUR foams (e.g., cell anisotropy, size, shape) [61,62,63]. Thus, the increase in λ should be explained by the influence of WS-based polyol on the cellular morphology of PUR foam. The replacement of petrochemical polyol with WS-based polyol led to less uniform cell structures with a lower number of closed-cells, which effectively increased the λ_r_ when compared to neat WS-0%, as was seen in SEM images (see Figure 6). It should be also noted that the value of λ is lower for the CO_2_ (0.015 W m^−1^K^−1^) than for the air (0.025 W m^−1^K^−1^), thus increasing the number of open-cell, the value of λ also increases. Furthermore, the density of the PUR foams increases with the weight ratio of WS-based polyol, which increases the heat transferred through the solid phase. The value of λ_s_ is additionally increased by the residual WS’s particles presented in the PUR structure, which might explain the relatively high coefficient of thermal conductivity of PUR foams containing WS-based polyol. Moreover, as presented in Figure 6, the structure of PUR foams containing WS-based polyol can be highly dense, since the hyperbranched polymer partially collapsed through the pores because of the flexible crosslinker. The reduced number of empty cells-filled with CO_2_ affects the heat transfer between the solid phase. The confirmation of this hypothesis might be found in the TGA results discussed in Section 3.9. For the WS-based foams, a marked increase of the char at high temperature (600 °C) with respect to the WS-0% was detected. This result could be ascribed to the presence of lignin in the walnut shell composition as well as to the more branched structure of WS-based foams, which results in the enhancement of thermal stability. 

### 3.7. Mechanical Properties

**Figure 8:**
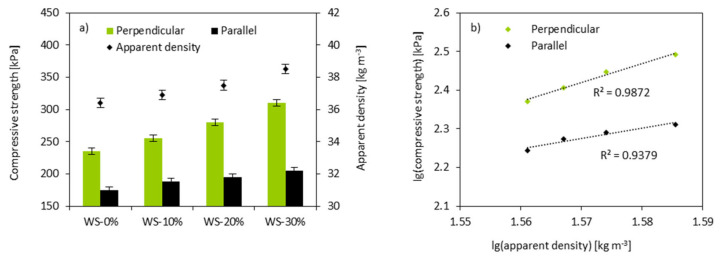
(**a**) dependence between σ_10%_ (measured perpendicular and parallel) and apparent density of PUR foams and (**b**) dependence between σ_10%_ and apparent density of PUR foams as a function of lg(compressive strength) = lg(apparent density).

The replacement of the petrochemical polyol with WS-based polyol affects the mechanical properties of PUR foams. As observed in Figure 8, when the weight ratio of WS-based polyol is increased from 10 to 30%, the compression strength (σ_10%_) is increased from 255 to 355 kPa. An analog behavior is observed for σ_10%_ measured in the transverse direction. Similarly, PUR foams containing 30% of WS-based polyol exhibit a higher value of σ_10%_ than WS-10% and WS-20%. Generally, the σ_10%_ of PUR foams has a positive correlation with their densities—the value of σ_10%_ of the PUR foam increases as its apparent density increases. The power-law has been evaluated to further examine the dependence between σ_10%_ and the apparent density of PUR foams. The results were expressed as lg(compressive strength)—lg(apparent density) correlation. The correlation coefficient of R is 0.998, which indicated that the hard segments formed by the lignin were significant when the amount of WS-based polyol exceeds 10%.

**Figure 9:**
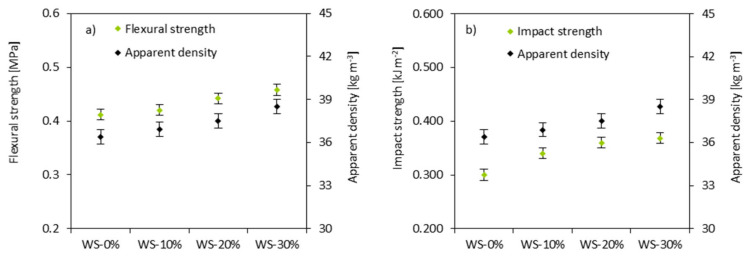
The dependence between (**a**) σ_f_, (**b**) σ_I_ and apparent density of PUR foams.

Figure 9 shows the flexural strength (σ_f_) and impact strength (σ_I_) as a function of increasing WS-based polyol loading. The value of σ_f_ measured for PUR foams increases with the weight ratio of WS-based polyol. For the PUR foams containing 30% of WS-based polyol, the corresponding increase is 10%. An analog dependence is observed in the case of σ_I_—the value of σ_I_ increases with the incorporation of WS-based polyol. In comparison to that of the WS-0%, the value of σ_I_ is increased by 13, 20, and 23% for WS-10%, WS-20%, and WS-30%, respectively. 

The improvement of mechanical properties with increasing the weight ratio of WS-based polyol can result from the formation of hard domains in the reaction between isocyanate and WS-based polyol. The existence of three-dimensional structures and aromatic rings in lignin structures may enhance a chain stiffness and the rigidity of foams, which results in the improved mechanical characteristics of PUR foams. Previous studies reported that the crack initiation energy is directly related to the nature of the matrix [64,65]. The energy increases with the WS-based polyol loading, which agrees with the increased ability of PUR foams to resist the fracture under the applied stress. Kosmela et al. [20] reported that the improvement of PUR foams containing marine-based polyol might result from the presence of the residual fillers particles, which are incorporated with the bio-polyol and act as reinforcing particles of composites. A similar explanation might be found in our study. When considering that the conversion of WS is ~87.2%, the residual WSs’ particles incorporated with WS-based polyol can inhibit the propagation of cracks and it can act as a load transfer medium in the PUR matrix. The large surface area of the WS particles interacts with the PUR matrix and reduces chain mobility, thereby increasing the rigidity of PUR foams and improving their mechanical properties. When the amount of the WS-based polyol is 30%, the properties of PUR foams meet the national standards established for rigid PUR foams (apparent density > 35 kg m^−3^, σ_10%_ > 300 kPa) [66]. 

### 3.8. Dynamic Mechanical Analysis (DMA) of PUR Foams

**Figure 10:**
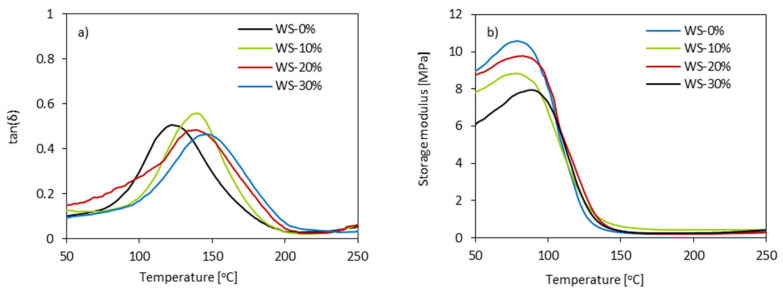
Dynamic mechanical analysis (DMA) results of PUR foams—(**a**) tanδ and (**b**) E’ in the function of temperature.

DMA was used to determine the viscoelastic properties of PUR foams. Figure 10 presents the results. The glass transition temperature (T_g_) of PUR foams has been considered as the maximum of tanδ peak in the function of the temperature (Figure 10a). It can be seen that all of the modified PUR foams possess one peak of maximum, which indicates that PUR foams can be classified as a homogeneous blend. PUR foams containing WS-based polyol have a higher value of T_g_ when compared with the neat WS-0%. With increasing the weight ratio of WS-based polyol from 10 to 30%, the value of T_g_ increases from 138 to 146%. Moreover, PUR foams containing WS-based polyol exhibit higher value of storage modulus (E’) (Figure 10b)—the greatest improvement in E’ is observed for WS-30%. Previous studies have shown that the dynamic-mechanical properties of PUR foams are affected by the cross-linking density as well as the aromaticity and aromaticity of PUR foams [67]. Because the isocyanate index is constant for all formulations, the increase in T_g_ can reflect increased aromaticity due to the presence of lignin. The confirmation of increased aromaticity might be found in the changes observed at ~100 °C, which refers to the hard segments of PUR and may act as chemical cross-link points of the PUR matrix. Therefore, increased values of T_g_ and E’ for PUR foams containing WS-based polyol indicate more restricted mobility of polymer chains, which results in the improvement of dynamic-mechanical properties of PUR foams. A similar effect was observed by Kosmela et al. [20]. PUR foams that were synthesized from marine biomass-based polyol (0–30 wt%) were characterized by higher T_g_ than PUR foams based on petrochemical-derived polyol, which was attributed to the more cross-linked structure of marine-based PUR foams.

### 3.9. Thermogravimetric Analysis (TGA) of PUR Foams

**Figure 11:**
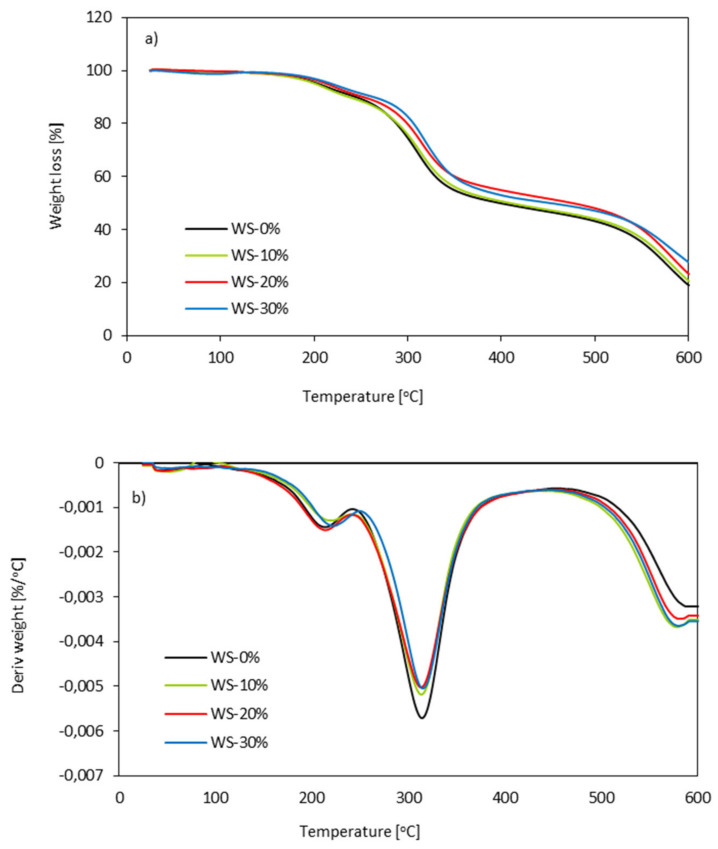
(**a**) Thermogravimetric Analysis (TGA) and (**b**) DTGA results.

TGA measurements were performed to evaluate the influence of WS-based polyol on the thermal characteristics of the modified PUR foams. Figure 11 shows the thermogravimetric (TGA) and derivative thermogravimetric (DTG) curves of PUR foams. The first stage of decomposition starts between 150 and 220 °C and it refers to the decomposition of isocyanate groups and pyranose rings [68,69]. In this study, the first degradation stage of PUR foams occurred at ~210 °C for WS-0% and 208, 210, 212 °C for WS-10%, WS-20%, and WS-30%. Increasing the concentration of WS-based polyol in the PUR foams, the cross-linking of PUR foam structure increases; therefore, more thermal energy is required to initiate the chain movements [70]. The second stage of degradation occurs between 300 and 350 °C and it refers to the decomposition of soft segments of PUR foams and decomposition of lignin [68]. As observed from DTG curves, the second stage of degradation for WS-0% is observed at ~308 °C. Insignificant improvement is observed for PUR foams with WS-based polyol. As compared to WS-0%, the temperature increases by 1–12 °C with increasing the weight ratio of WS-based polyol from 10 to 30%. Such an improvement can result from the enhanced crosslink density with an increase in the weight ratio of WS-based polyol, which reduced the mobility of the polymeric chains and difficulty of heat transferred into PUR foams [70].

The last degradation stage occurs between 500 and 600 °C and it refers to the decomposition of WS-based components—cellulose, hemicellulose, and lignin [71]. With increasing the weight ratio of WS-based polyol the mass loss is increased due to a higher concentration of WS-based polyol and partial miscibility of the aromatic hard and soft segments [70]. Moreover, with increasing the weight ratio of WS-based polyol the amount of char residue at 600 °C increases. For WS-0%, the char residue is 20.8%, while, for PUR foams with WS-based polyol, the amount of char residue increases from 23.1% (for WS-10%) to 29.7 (for WS-30%). The rigid structure of lignin and the cross-linked network structure formed during the preparation of PUR foams can somewhat increase the heat resistance of the PUR foams with increasing the weight ratio of WS-based polyol [72]. Similar results have also been reported in previous studies [73,74].

### 3.10. Color Analysis, Water Uptake and Dimensional Stability of PUR Foams

**Figure 12:**
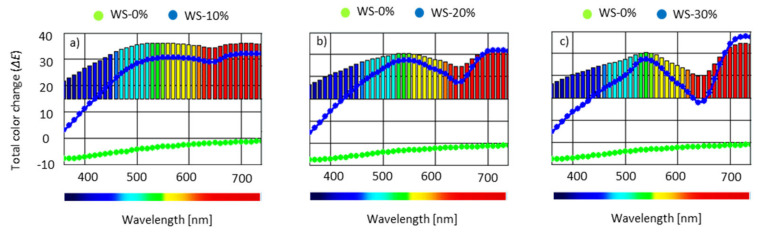
Color characteristic of (**a**) WS-10%, (**b**) WS-20%, and (**c**) WS-30% in comparison to WS-0%.

The influence of WS-based polyol on color characteristics of PUR foams was optically determined. Table 5 and Figure 12 present the results of the color characteristics. Here, ΔE* determines color difference, L* determines the color from black to white (from 0 to 100), +a* to −a determines red and green shades, while +b* to −b* determines yellow and blue shades. In general, it is observed that the color of PUR that is modified with WS-based polyol has changed from medium brown (for WS-10%) to dark brown (for WS-30%). Based on ΔE* results, the most pronounced effect is observed for WS-30%. For WS-10% and WS-20%, the value of ΔE* oscillates in the range of ~30, while, for WS-30%, the value of ΔE* increases to ~40. A similar trend is observed for the brightness (L*)—with increasing the WS-based polyol content, the color of PUR foams becomes more intense, which is confirmed by the lower value of *L. When comparing to WS-0%, PUR foams containing WS-based polyol have greater values of a* and b*. This indicates that the incorporation of WS-based polyol results in obtaining PUR foams with more intense red and yellow shades.

**Figure 13:**
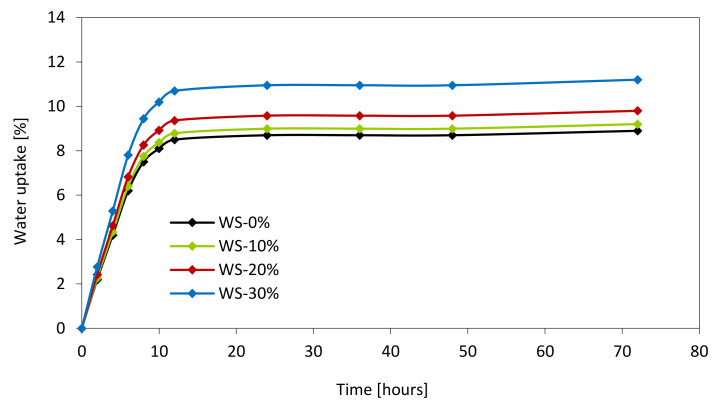
Water uptake of PUR foams as a function of time.

Figure 13 presents the results of water uptake. The water uptake was measured as the percentage weight gain after 72 h of measurement. Based on the results, it can be stated that the incorporation of WS-based polyol increases the water uptake of PUR foams. In all cases, a significant rise in water intake was observed during the first 8 h. Over the next few hours, the gradual stabilization in water intake was observed. Previous work has shown that water uptake depends on cellular morphology [75]. As previously presented, by increasing the weight ratio of the WS-based polyol, the number of open cells increases. Since the open pores can absorb more water, the enhanced water uptake is observed. Moreover, PUR foams synthesized from WS-based polyol are characterized by the structure with smaller cells that possess greater surface area and can absorb more water [76]. In comparison to the neat WS-0%, the water uptake of WS-10%, WS-20%, and WS-30% increases by 2, 10, and 25%; however, the obtained results are still in line with the commercial standards [77].

**Figure 14:**
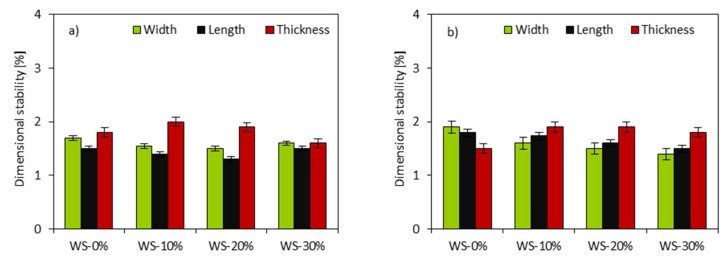
Dimensional stability of PUR foams measured at (**a**) +70 °C and (**b**) −20 °C.

The dimensional stability of PUR foams was evaluated. The results that are presented in Figure 14 indicate that the replacement of petrochemical polyol with WS-based polyol has no influence on the dimensional stability of analyzed PUR foams. In all cases, the linear changes are insignificant and no relationship is observed between the results. The dimensional stability results may result from experimental errors. Besides this, all of the modified PUR foams are in the line with commercially acceptable standards, which require the linear changes below 3% after the conditioning at 70 °C, and below 1% after the conditioning at −20 °C [78].

## 4. Conclusions

This study aimed to examine rigid polyurethane (PUR) foams properties that are based on WS-based polyol. FTIR results revealed that the liquefaction of walnut shells was successfully performed. The three types of PUR foams were synthesized by replacement of 10, 20, and 30 wt% of the petrochemical polyol with WS-based polyol. The impact of WS-based polyol on the cellular morphology, mechanical, thermal, and insulating characteristics of PUR foams was examined. The produced PUR foams had apparent densities from 37 to 39 kg m^−3^, depending on the weight ratio of WS-based polyol. PUR foams that were obtained from WS-based polyol had improved mechanical characteristics when compared with PUR foams derived from the petrochemical polyol. PUR foams produced from WS-based polyol showed compressive strength from 255 to 310 kPa, flexural strength from 420 to 458 kPa, and impact strength from 340 to 368 kPa. The foams produced from WS-based polyol exhibited less uniform and cell structure than foams derived from the petrochemical polyol. Additionally, with the increasing concentration of WS-based polyol, the number of closed-cells slightly decreased. The thermal conductivity of the PUR foams ranged between 0.026 and 0.032 W m^−1^K^−1^, depending on the concentration of WS-based polyol. The addition of WS-based polyol had no significant influence on the thermal degradation characteristics of PUR foams. The maximum temperature of thermal decomposition was observed for PUR foams with the highest loading of WS-based polyol.

## Figures and Tables

**Table 1:** Formulations of polyurethane (PUR) foams.

Component	WS-0%	WS-10%	WS-20%	WS-30%
	Parts by weight (wt%)			
STEPANPOL PS-2352	100	90	80	70
PUROCYN B	160	160	160	160
Kosmos 75	6	6	6	6
Kosmos 33	0.8	0.8	0.8	0.8
Tegostab B8513	2.5	2.5	2.5	2.5
Water	0.5	0.5	0.5	0.5
Pentane/cyclopentane	11	11	11	11
WS-based polyol	0	10	20	30

**Table 2:** Properties of walnut shells (WS)-based polyol and commercial petrochemical polyol.

	Viscosiy [mPa s]	Hydroxyl Number [mg KOH/g]	Water Content [%]	Molecular Weight (M_w_) [Da]	Liquefaction Ratio [%]
WS-based polyol	2550	340	0.8	420	87.2
Petrochemical polyol	2000–4500	230–250	0.2	468	N/D

**Table 3:** Influence of WS-based polyol on processing times, maximum temperature, and final height of PUR foams.

	WS-0%	WS-10%	WS-20%	WS-30%
Start time	55 ± 3	61 ± 1	64 ± 2	70 ± 3
Growth time	430 ± 8	510 ± 9	515 ± 9	590 ± 10
Tack-free time	355 ± 9	420 ± 8	450 ± 9	520 ± 8
T_max_	150	125	120	105
Height	205	180	160	145

**Table 4:** Cell size, closed-cell content, apparent density, and thermal conductivity of PUR foams.

	WS-0%	WS-10%	WS-20%	WS-30%
Cell size [µm]	345 ± 9	320 ± 8	310 ± 6	280 ± 8
Closed-cell content [%]	92.1	89.3	86.4	80.2
Apparent density [kg m^−3^]	36 ± 1	37 ± 1	38 ± 2	39 ± 1
Thermal conductivity [W m^−1^ K^−1^]	0.024	0.026	0.029	0.032

**Table 5:** Color characteristics of PUR foams—brightness (L*), red-green shades (a*), yellow-blue shades (a*), total color difference (ΔE*).

Sample Code	Colorimetric Parameters			
	L*	a*	b*	ΔE*
WS-0%	60.25	22.45	−5.10	0
WS-10%	45.10	35.50	−2.10	29.85
WS-20%	40.20	40.20	−0.60	29.94
WS-30%	24.25	48.50	1.40	39.73
